# Identification of Stable and Multiple Environment Interaction QTLs and Candidate Genes for Fiber Productive Traits Under Irrigated and Water Stress Conditions Using Intraspecific RILs of *Gossypium hirsutum* var. MCU5 X TCH1218

**DOI:** 10.3389/fpls.2022.851504

**Published:** 2022-04-18

**Authors:** Narayanan Manikanda Boopathi, Gopal Ji Tiwari, Satya Narayan Jena, Kemparaj Nandhini, V. K. I. Sri Subalakhshmi, Pilla Shyamala, Babita Joshi, Nallathambi Premalatha, S. Rajeswari

**Affiliations:** ^1^Department of Plant Biotechnology, CPMB&B, Tamil Nadu Agricultural University, Coimbatore, India; ^2^Plant Molecular Genetics Laboratory, CSIR-National Botanical Research Institute, Lucknow, India; ^3^Department of Cotton, CPBG, Tamil Nadu Agricultural University, Coimbatore, India; ^4^Academy of Scientific and Innovative Research (AcSIR), Ghaziabad, India

**Keywords:** drought, productive traits, genetic map, SNP, QTL, upland cotton, intra-specific cross

## Abstract

Cotton productivity under water-stressed conditions is controlled by multiple quantitative trait loci (QTL). Enhancement of these productivity traits under water deficit stress is crucial for the genetic improvement of upland cotton, *Gossypium hirsutum*. In the present study, we constructed a genetic map with 504 single nucleotide polymorphisms (SNPs) covering a total span length of 4,416 cM with an average inter-marker distance of 8.76 cM. A total of 181 intra-specific recombinant inbred lines (RILs) were derived from a cross between *G*. *hirsutum* var. MCU5 and TCH1218 were used. Although 2,457 polymorphic SNPs were detected between the parents using the CottonSNP50K assay, only 504 SNPs were found to be useful for the construction of the genetic map. In the SNP genotyping, a large number of SNPs showed either >20% missing data, duplication, or segregation distortion. However, the mapped SNPs of this study showed collinearity with the physical map of the reference genome (*G. hirsutum* var.TM-1), indicating that there was no chromosomal rearrangement within the studied mapping population. RILs were evaluated under multi-environments and seasons for which the phenotypic data were acquired. A total of 53 QTL controlling plant height (PH), number of sympodial branches, boll number (BN), and boll weight (BW) were dissected by QTL analysis under irrigated and water stress conditions. Additionally, it was found that nine QTL hot spots not only co-localized for more than one investigated trait but were also stable with major QTL, i.e., with > 10% of phenotypic variation. One QTL hotspot on chromosome 22 flanked by AX-182254626–AX-182264770 with a span length of 89.4 cM co-localized with seven major and stable QTL linked to a number of sympodial branches both under irrigated and water stress conditions. In addition, putative candidate genes associated with water stress in the QTL hotspots were identified. Besides, few QTL from the hotspots were previously reported across various genetic architects in cotton validating the potential applications of these identified QTL for cotton breeding and improvement. Thus, the major and stable QTL identified in the present study would improve the cotton productivity under water-limited environments through marker-assisted selection.

## Introduction

Owing to its affordability, appearance, and natural comfort in apparel, cotton (*Gossypium* spp.) is the most preferred economic crop globally. More than 80% of the top ten cotton cultivating zones are in developing countries, and the cotton export value of these zones exceeded USD 30 billion in 2017 (^1^ accessed on 24 December, 21). Thus, cotton promotes economic development and offers a key source of livelihood for millions of farmers in the world’s top three cotton exporters, North America, Central Asia, and West Africa (Jans et al., 2021).

India had a total cotton area of 13.48 million ha, accounting for 42.07% of the world’s cotton area (32.04 million ha). Particularly, the textile belt of South India alone has more than 2,000 large and small cotton mills that manufacture blended yarns and cotton with the export revenue of more than USD 3,757 million, and it is predicted that the expected flow of investment in South India in various textile activities during next 5 years will be USD 14,279 million (^2^ accessed on 24 December, 21). Consequently, there is a continuous demand for cotton products in India and across the world, but fiber yield is challenged by the unpredictable climate change in all the cotton-producing countries (Niu et al., 2018). For example, more than 60% of cotton farmed in India is rainfed (Gutierrez et al., 2015), where erratic rainfall patterns severely limit lint output. Similarly, despite having one of the most efficient cotton industries in the world in the terms of water use, a recent Australian Bureau of Statistics report released on 14 May 2021 stated that climatic caprices significantly reduced Australia’s cotton area (^3^ accessed on 24 December, 21). Cotton productivity under these rainfed conditions largely depends on the timing, distribution, and quantity of monsoon rains at the different growth phases of cotton, and hence water stress limits lint productivity with different magnitudes, depending on its occurrence and severity. In general, high temperatures, below-average rainfall, and limited water availability in rainfed cotton have severely limited cotton output in recent years. There is an urgent need to develop a cotton cultivar that is more productive in water-stressed settings.

Among the six allotetraploid species (2*n* = 4×, AADD), *Gossypium hirsutum* L., contributes >90% of the worldwide cotton production (Shim et al., 2018). Conventional breeding efforts to improve the fiber yield in this species under water-limited environments have shown slow progress as these traits are more complex and governed by multiple genetic and environmental factors (Tan et al., 2018). Furthermore, the negative relationship between fiber quality and yield component characteristics impedes the simultaneous enhancement of yield and fiber quality traits in *G. hirsutum* (Abdelraheem et al., 2018). Thus, before attempting to improve these complex traits under rainfed conditions, the genetics underlying fiber production and quality attributes (such as, the pleiotropic impact and functional genes that govern trait) must be dissected by quantitative trait loci (QTL) mapping.

Among the target traits to be dissected, it is imperative to dissect the QTL for optimum plant height (PH), zero or minimum monopodial branch length, number of bolls, and boll weight, as these traits are key in introducing the mechanical harvesting, which is the need of the hour (owing to the permanent paucity of labor for boll picking). The cotton PH of 80–120 cm is strongly related to the yield under mechanical harvesting (Yan et al., 2019), and the desired form of a cotton cultivar is a compact architecture with short fruit internodes and tightly packed cotton bolls. Furthermore, such cotton designs stimulate the use of less plant growth regulators, are resistant to lodging, and adapt themselves to the dense planting and automated harvesting (Wen et al., 2021). Advances in different molecular marker tools and strategies used to dissect the complex traits genetically have enabled fast-track molecular breeding efforts in several crops even in cotton (Boopathi, 2020). A large number of QTL associated with agronomic, yield, and particular fiber quality traits were identified, and available as valuable databases on cotton QTL, such as CottonQTLdb (Said et al., 2015) and CottonGen (Yu et al., 2021). Such attempts may allow the identification of stable QTLs’ co-localization across *G. hirsutum* genetic backgrounds, validating the found QTL for future use in marker-assisted selection (MAS) for the efficient and simultaneous augmentation of yield and fiber quality traits. The use of MAS to pyramid several QTLs that affect numerous desired target characteristics enhanced the selection efficiency among breeding populations with varied genetic origins. Recent studies (Abdelraheem et al., 2021; Guo et al., 2021; Wang H. et al., 2021; Wang N. et al., 2021; Zhu et al., 2021) demonstrated the necessity of dissecting complicated fiber traits using QTL mapping and the potential of MAS in the cotton breeding program.

Though India has a long history of cotton breeding efforts, only a few reports have employed molecular markers to analyze the genetic purity of seed lots (Selvakumar et al., 2010) to examine the level of genetic diversity and linkage disequilibrium in cotton subpopulations (Jena et al., 2011) and to genetically dissect cotton fiber traits (Boopathi et al., 2015; Kumar et al., 2019; Shukla et al., 2021). An array of QTL, genes, and gene products are specifically involved in drought response in cotton (Deeba et al., 2012), but identifying which are most useful for cotton breeding in the genetic improvement of drought tolerance in the regional breeding program has remained a significant challenge, owing to the unique characteristics of the target population of environments under rainfed conditions. As a result, anticipating the future uses of MAS in cotton, it is critical to begin the QTL mapping utilizing various parental lines and introgression of important and stable QTL for target characteristics by MAS for the genetic improvement of cotton in water-limited settings.

While selecting the donors for the QTL mapping of drought tolerance and fiber yield/quality traits, *G. arboreum* and *G. barbadense*, respectively, would be the best choice owing to their superior trait values. However, the transfer of other undesirable agronomic traits through linkage drags and problems associated with cross ability, return toward the genotype of one parent (segregation distortion), and the suppression of recombination greatly limit the progress and made the interspecific crossing program as a challenging task (Boopathi and Hoffmann, 2016). As a result, breeding initiatives are mostly focused on intraspecific *G. hirsutum* cross combinations to develop superior lint production and fiber quality attributes at a lower cost. On the other hand, the lack of polymorphism created across intraspecific *G. hirsutum* lines using breeder-friendly second-generation molecular markers, such as simple sequence repeats (SSRs) impedes the efficiency of QTL mapping (Shen et al., 2005). Through these efforts, even a consensus map derived from several intra-specific maps from different mapping populations encompassed just 31% of the cotton genome (Ulloa et al., 2005).

Alternatively, recent advancements in high throughput genotyping systems with enhanced the effectiveness in producing polymorphism among closely related individuals have made the single nucleotide polymorphism (SNP) a widely used and popular marker in plant translational quantitative genetics (Hulse-Kemp et al., 2015). With the improvement of next-generation sequencing technology, numerous methodologies have been used to identify a significant number of polymorphic SNPs in cotton, which might be useful for high-density mapping and more efficient QTL analysis. As a result, it is particularly desirable to use third-generation markers, such as SNPs to improve the detection of polymorphic loci between closely related *G. hirsutum* parental lines.

Considering the above, this present study was designed to identify QTL for various yield traits under irrigated and water-limited environments in *G. hirsutum* using SNP markers. We selected *G. hirsutum* parents, MCU5 and TCH1218, differing distinctly for drought tolerance and various other traits to develop the recombinant inbred lines (RILs) and evaluated them under irrigated and water-stressed field conditions for the identification of fiber yield related QTLs by employing 50K SNP custom array with Axiom technology (Affymetrix).

## Materials and Methods

### Plant Materials

Recombinant inbred lines derived from the intra-specific cross between *G. hirsutum* var. MCU5 and TCH1218 were used in this study. The F_1_ was made at Tamil Nadu Agricultural University (TNAU), Coimbatore, in the year 2006 and forwarded to F_11_ using the single seed descent method. In total, 181 RILs from the F_10_ generation were employed in the construction of the genetic map. Data on PH (cm), number of sympodial branches, number of bolls, and boll weight (g) obtained from the phenotypic evaluation of F_7_ to F_11_ of those same RILs were used for QTL analysis. The female parent MCU5 is a multiple cross derivative cultivated widely in South India as it possesses medium staple fiber (29 mm) and can be spun up to 70s. Besides, it has a 34% ginning outturn and produces 1,850 kg seed cotton yield per ha; however, it cannot withstand water stress at the flowering phase (Boopathi et al., 2014). On the other hand, the male parent, TCH1218, has relatively better drought tolerance but low fiber yield and quality characters than MCU5 (Boopathi et al., 2014). Our historical breeding effort has shown that MCU 5 has long-staple cotton with a good yield, while TCH 1218 is a good combiner.

### Phenotypic Data

All the plant materials (181 RILs and two parental lines) were evaluated in randomized block design with two replications at different locations and seasons (the details of testing environments are provided in Table 1). The plant-to-plant distance was 45 cm, while the row-to-row distance was 90 cm, and 13 plants were maintained in each row. Regular crop husbandry measures were followed to ensure a healthy crop. To collect phenotypic data, three plants were selected randomly per replication on 115 days after sowing (DAS), and information was collected on different yield parameters from each experiment as detailed in Table 1. RILs were evaluated under field water stress conditions in two different water-stress environments: one with managed water stress (by withholding irrigation water after 45th day) at Maize Research Station (MRS), TNAU, Vagarai, and another under purely rainfed conditions at Cotton Research Station (CRS), TNAU, Veppanthattai and Agricultural Research Station (ARS), TNAU, Aruppukottai. Regular agricultural practices were used to control weeds, diseases, pests, and fertilizers were applied at sufficient levels to ensure that yield potential was not limited by any factor other than water. At MRS (T2 in Table 1), water stress was imposed by withholding water from 45th DAS, whereas the irrigated control plots received water at routine intervals. At CRS T3 and T7 (Table 1) and ARS T6 (Table 1), the RILs were grown purely under rainfed conditions, and there was no rain after 51 and 57 DAS at CRS during 2012 and 2018, respectively, and 58 DAS at ARS during 2018.

**TABLE 1:** Details on testing environments used for the phenotyping of different recombinant inbred line (RIL) generations derived from MCU5 and TCH1218.

Trial ID	F_x_	Year	Location and status of phenotyping	Elevation above mean sea level (m)	Coordinates (Latitude Longitude)	Soil	Average weather parameters during Cropping period			Phenotyping data collected
							Temperature Maximum (°C) Minimum (°C)	Rainfall (mm)	Relative humidity (%)	
T1	F_7_	Aug., 2011–Dec., 2011	Maize Research Station (MRS),Vagarai; (Under Irrigated control conditions (IC))	254	10.5844° N, 77.5727° E	Black, clay loam	32.5 24.0	814	71.6	PH, SYM, and BN
T2	F_7_	Aug., 2011–Dec., 2011	MRS, Vagarai; (Under water stress conditions (WS))	254	10.5844° N, 77.5727° E	Black, clay loam	32.5 24.0	814	71.6	PH, SYM, and BN
T3	F_8_	Aug., 2012–Dec., 2012	Cotton Research Station (CRS),Veppanthattai; (Under rainfed conditions (RF))	149	11.3794° N, 78.7297° E	Black, clay	31.4 27.5	662	69.4	PH, SYM, BN, and BW
T4	F_9_	Sep., 2013–Jan., 2014	Eastern Block, TNAU, Coimbatore; (IC)	432	11.0168° N, 76.9558° E	Mediumblack, sandy clay loam	28.8 22.0	564	75.2	PH, SYM, BN, and BW
T5	F_10_	Aug., 2018–Dec., 2018	Department of Cotton, TNAU, Coimbatore; (IC)	432	11.0168° N, 76.9558° E	Red sandy clay loam	27.6 21.5	481	76.2	PH, SYM, BN, and BW
T6	F_10_	Aug., 2018–Dec., 2018	Agricultural Research Station, Aruppukottai; (RF)	97	9.5139° N, 78.1002° E	Black, clay	38.2 22.1	616	71.4	PH, SYM, BN, and BW
T7	F_10_	Aug., 2018–Dec., 2018	Cotton Research Station (CRS), Veppanthattai; (RF)	149	11.3794° N, 78.7297° E	Black, clay	32.3 26.1	580	67.8	PH, SYM, BN, and BW
T8	F_11_	Aug., 2019–Dec., 2019	Department of Cotton, TNAU, Coimbatore; (IC)	432	11.0168° N, 76.9558° E	Red sandy clay loam	28.6 20.3	501	75.8	PH, SYM, BN, and BW

*F_x_: Filial generation of MCU5 × TCH1218; PH, plant height (cm); SYM, number of sympodial branches; BN, number of bolls per plant; BW, boll weight (g).*

Standard descriptive statistical analysis to examine the significance of the difference in the investigated traits between two parents and among population and the estimation of Pearson’s correlation coefficient was done using Calculator Soup^®4^ and Minitab^®^ 19^5^, respectively.

### DNA Isolation and Genotyping

Genomic DNA was extracted from the young leaves of mapping population and parents using a modified cetyltrimethylammonium bromide (CTAB) method (Shukla et al., 2021). SNP genotyping was performed using the Gene Titan Multi-channel instrument (Thermo Scientific) facility at M/s Imperial life Sciences, New Delhi, with the Cotton 50K SNP array chip. DNA samples were first processed to the amplification, fragmentation, precipitation, and re-suspension and then hybridized to the chip using the Affymetrix reagents. The arrays were scanned with the Gene Titan MC and automated allele calling, and the quality assessment of called genotypes was done with Genotyping Console*™* Software (GTC) with a new Axiom Genotyping Algorithm v1 (Axiom GT1). Consequently, the raw hybridization intensity data were processed for clustering and genotype calling with Affymetrix^®^ GTC (v4.2), and those data with Dish Quality Control (DQC) value <0.82 and call rate <0.97 were excluded from the further genotyping analysis. To finish, GTC was further processed using APT (v1.19.0) and classified those SNPs into six major classes. The raw genotyping data were again filtered by selecting only those co-dominantly segregating SNPs with <20% missing genotyping data (Hulse-Kemp et al., 2015). Subsequently, the genotyping data were transformed into a mapping data format. The probe sequences (10–20 bp overlapping to SNP) were also mapped on reference genome *G. hirsutum* cultivar TM-1 (ASM98774v1), and the SNPs were assigned to the particular chromosomes. The same data were used as anchoring information while preparing the input file for the mapping software while performing chromosome assignments.

### Genetic Map Construction

A total of 522 polymorphic and segregating SNP markers were used to construct the genetic map using ‘IcImapping v4.0’ (Meng et al., 2015), and the genetic map distance (centiMorgan, cM) was calculated using the Kosambi mapping function. The threshold for logarithm of odds (LOD) score was fixed at five, revealing 26 linkage groups. The exact ordering of the SNPs across chromosomes was done using RECORD, and Rippling was also executed for fine-tuning the order of markers by the sum of adjacent recombination frequencies (COUNT) with a window size of five. If there were more than three consecutive adjacent markers in the genetic map with a significance level 0.001 < *p* < 0.05, it was taken as segregation distortion, and its distribution on the map was also analyzed (Coulton et al., 2020).

### Quantitative Trait Loci Analysis for Production Traits

Composite interval mapping (CIM) with forward regression, in a window size of 10 cM and 5 background control markers at a walking speed of 2 cM, was employed for QTL analysis using Windows QTL Cartographer 2.5 (Wang et al., 2012). The threshold LOD of each trait QTL was determined with the trait threshold calculated with a permutation of 10,000, and multiple QTL peaks detected within 10 cM of each other were regarded as a single QTL. To calculate the effects of additive QTL in multiple environments, the MET functional module of QTL IciMapping v4.0 was employed by combined mapping analysis under multi-environment with 3.00 permutation tests’ LOD cut-off values.

### Collinearity and Recombination Hotspot Analysis

All the SNP markers used to construct the genetic map reported in this study were aligned to the physical map of the upland cotton genome (TM-1 Genome NAU-NBI Assembly v1.1 and Annotation v1.1 database) through Basic Local Alignment Search Tool (BLAST). CIRCOS 0.66 with default parameters was employed to compare each investigated SNP marker’s genetic and physical positions collinearity. The recombination hotspot (RH) was estimated by inferring the recombination rate of investigated SNPs. If the genetic distance between adjacent SNPs was higher than 20 cM/Megabase, the genomic region between those markers was viewed as RH (Zhang J. et al., 2015).

### Major and Stable Quantitative Trait Loci and Identification of Putative Candidate Genes

Quantitative trait loci co-localized at least in two trials with ≥10.0 R^2^ for at least one trait were depicted as major and stable QTL, and they were selected to scan for candidate genes. In general, a line with at least 10% higher variation than the parents for the target traits is considered an improved line; hence ≥10.0 R^2^ is depicted as a major QTL in this study. The sequences of SNP markers flanking the confidence intervals (*CI*s) of the QTL were aligned back to the physical sequence of the upland cotton genome database (Zhang J. et al., 2015). Based on the position of these flanking markers, all the genes within the target QTL were identified as candidate genes. Gene ontology (GO) grouping was employed to categorize the identified candidate genes, and pathways correlated to the candidate genes were also discovered using Kyoto Encyclopaedia of Genes and Genomes (KEGG) analysis (Charmpi and Ycart, 2015).

## Results

### Phenotypic Variation for Parents and Recombinant Inbred Lines

The differences noticed for the investigated phenotypic traits between parents and the variation among the investigated RILs are provided in Table 2. As expected, the elite parent MCU5 performed well under the irrigated conditions, whereas TCH1218 had produced substantially good yield under both managed water stress and rainfed conditions by having higher values in the relative performance of investigated traits (significant at the 0.05 probability level) (Table 2). Transgressive segregation was observed in the RIL population for all the investigated traits under irrigated and water-stressed conditions. However, PH and boll weight have shown substantially skewed distribution as their skewness values were greater than +1 or lower than −1 (Table 2). All possible kinds of distribution, such as fairly symmetrical (skewness −0.5 and 0.5), moderately skewed skewness (skewness −1 and −0.5 or between 0.5 and 1), and highly skewed (skewness less than −1 or greater than 1) distribution of the traits examined in this study were noticed (Table 2). As the calculated kurtosis values for all the investigated traits were greater than zero (Table 2), it can be concluded that each trait distribution has a heavier tail (leptokurtic distribution). Substantial variations (minimum and maximum values of each trait) due to genotype differences were reported for all the investigated traits among the RILs. However, the relative proportions of variance varied from one trait to another, and there was low to moderate heritability noticed for the investigated traits (Table 2). The correlation between investigated traits measured from all the trials (T1–T8) was also evaluated (Table 3). Although not in all the trials, the majority of correlations have shown a highly significant positive correlation between yield (BN and BW) and growth parameters (PH and SYM) both under irrigated and water-stressed environments, indicating that genetic improvement BN and BW would likely be accompanied by the improvement of PH and SYM. However, significant negative correlations were also observed (Table 3), which showed that the simultaneous improvement of investigated traits could not be done for all the cotton-growing regions. In general, the correlation between the investigated traits increased dramatically under rainfed conditions (T3 and T7); however, mixed trends were noticed in one rainfed environment, T6 (Table 3). On the other hand, under managed water stress conditions (T2), a reduced level of correlation between the measured traits was noticed when compared with the traits measured from irrigated conditions (T1) (Table 3).

**TABLE 2:** Phenotypic variation and descriptive statistical analysis of data on yield traits obtained from RILs derived from MCU5 and TCH1218 across the environments and seasons.

Trait	Trial*	MCU5 Mean	TCH1218 Mean	RILs							Broad sense heritability
				Mean	SD	CV	Minimum	Maximum	Skewness γ_1_	Kurtosis β_2_	
Plant Height	T1	116.99	124.66	100.03	27.57	0.27	26.00	167.33	−1.51	7.08	0.58
	T2	92.16	101.00	88.13	31.07	0.35	10.00	159.33	−1.35	5.73	0.55
	T3	42.00	60.58	95.02	43.07	0.45	21.00	157.00	−1.01	3.00	0.45
	T4	125.00	132.00	106.55	27.89	0.26	66.33	161.00	−1.57	8.11	0.54
	T5	102.95	100.25	112.82	29.52	0.26	10.10	143.19	−2.85	10.85	0.45
	T6	83.00	87.00	84.03	13.08	0.15	46.50	122.83	−0.02	3.10	0.43
	T7	107.50	137.00	117.55	20.75	0.17	69.33	185.00	0.45	2.84	0.58
	T8	109.90	123.40	120.32	10.47	0.08	92	143.9	−0.23	2.48	0.41
Number of Sympodia	T1	11.87	10.25	14.23	6.04	0.42	2.00	28.5	−0.30	2.72	0.50
	T2	15.49	39.99	17.30	7.42	0.42	6.5	51.33	−0.05	5.22	0.55
	T3	1.83	3.66	5.21	2.48	0.47	1.00	12.00	−0.13	3.21	0.44
	T4	4.00	3.66	2.23	0.85	0.38	1.00	5.00	0.50	3.25	0.48
	T5	11.40	11.40	15.32	8.11	0.52	1.00	9.05	7.82	9.93	0.47
	T6	7.5	10.58	10.57	1.71	0.16	6.83	19.24	0.62	4.93	0.58
	T7	9.33	19.67	14.34	3.18	0.22	5.67	24.00	0.18	2.76	0.47
	T8	10.90	12.90	15.21	1.51	0.09	11.2	18.8	−0.27	3.09	0.44
Number of Bolls per Plant	T1	10.80	9.10	6.80	3.70	0.54	1.33	25.00	1.37	6.96	0.55
	T2	2.83	7.33	4.39	2.44	0.55	0.66	11.33	0.26	2.86	0.58
	T3	2.66	5.16	13.03	6.62	0.50	2.50	27.00	−0.46	2.85	0.41
	T4	16.67	15.33	17.61	5.99	0.34	7.67	48.67	0.18	7.84	0.48
	T5	24.00	21.00	21.99	4.60	0.20	11.0	30.00	−0.52	2.48	0.49
	T6	11.92	16.25	19.03	4.67	0.24	8.67	43.17	0.64	5.26	0.45
	T7	17.00	40.33	23.06	6.70	0.29	8.33	45.00	0.64	3.45	0.58
	T8	13.60	12.60	15.17	2.95	0.19	8.80	22.97	0.19	2.78	0.57
Boll Weight	T3	2.65	5.75	5.19	2.03	0.39	3.00	8.80	−1.55	4.93	0.44
	T4	4.73	5.00	4.19	1.14	0.27	2.50	10.67	−0.55	12.49	0.44
	T5	4.00	3.20	2.93	0.67	0.22	1.20	4.30	−0.002	2.35	0.58
	T6	4.65	4.90	3.78	0.75	0.19	1.67	5.60	0.12	2.92	0.56
	T7	3.47	4.51	3.67	0.63	0.17	2.15	6.13	0.12	3.25	0.41
	T8	4.45	4.46	4.63	0.60	0.13	2.47	6.08	−0.34	3.83	0.59

*As provided in Table 1; SD, standard deviation; CV%, coefficient of variation.

**TABLE 3:** Correlation coefficients among the investigated traits measured from RILs used in this study.

	PH	SYM	BN
**T1**			
SYM	0.613		
BN	0.567	0.509	
**T2**			
SYM	0.442		
BN	0.454	0.260	
**T3**			
SYM	0.794		
BN	0.825	0.845	
BW	0.606	0.512	0.553
**T4**			
SYM	0.211		
BN	0.255	0.057^$^	
BW	0.098^$^	0.052^$^	0.192
**T5**			
SYM	0.124		
BN	0.104^$^	0.875	
BW	−0.284	0.853	0.770
**T6**			
SYM	0.040^$^		
BN	0.405	0.753	
BW	−0.155	0.864	0.594
**T7**			
SYM	0.941		
BN	0.947	0.989	
BW	0.889	0.966	0.936
**T8**			
SYM	0.595		
BN	0.061^$^	0.391	
BW	−0.024^$^	−0.034^$^	−0.069^$^

*All the below mentioned Pearson’s correlation coefficient has p < 0.05 except those marked with ^$^, which has p > 0.05.*

*T1 to T8, PH, SYM, BN, and BW: as mentioned in Table 1.*

### Genetic Map Construction With Cotton 50K Single Nucleotide Polymorphisms Array

Affymetrix’s Axiom custom designed Cotton 50K SNP array (unpublished) was used to the SNP typing of both parents and 181 RILs. This resulted a total of 2,457 polymorphic SNPs between the two parents and those polymorphic markers were analyzed for their segregation in the mapping population. The initial attempt of binning of these 2,457 loci by excluding those SNPs with more than 20% missing data (totally there were 17 such markers) resulted in 2,440 polymorphic SNPs, which were further analyzed with χ^2^ test to determine if the allele frequency was deviated from the expected segregation ratio (1:1). Among the selected 2,440 polymorphic loci, 1,913 loci showed segregation distortion (*p* < 0.05), and only 527 loci followed the perfect Mendelian ratio. It was also noticed that among 1,913 distorted loci, 1,186 were in favor of TCH1218 alleles, and 727 loci were biased toward MCU5 alleles. Further, it has been found that among the 527 loci that were segregated in the RILs, and five were found to be duplicated.

**FIGURE 1:**
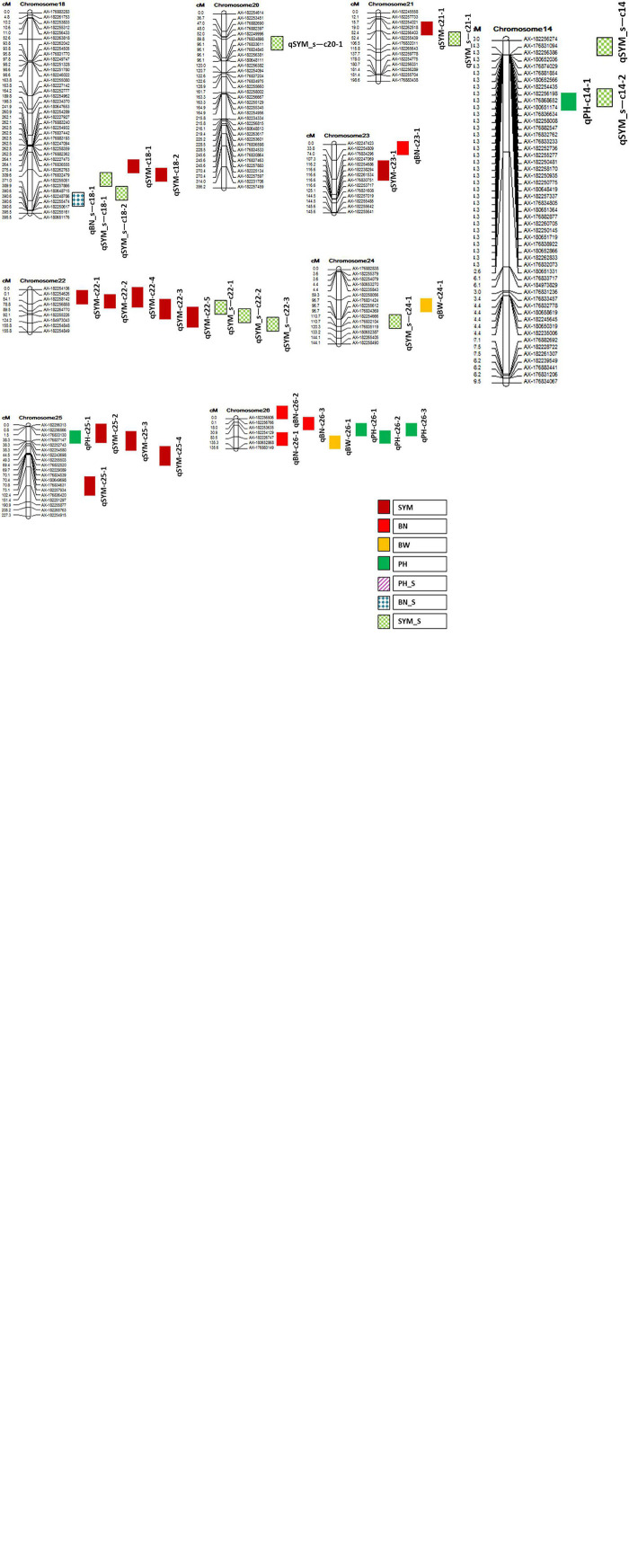
Linkage map showing the relative positions of quantitative trait loci (QTL) identified in this study. QTL nomenclature is as indicated in Table 6.

Therefore, only 522 polymorphic SNPs were taken for the genetic mapping, out of which 504 were mapped on 26 AD linkage groups (LGs) or cotton chromosomes (as 18 SNPs were unlinked), and the resultant genetic map represented the genetic span length of 4,416 cM. The At sub-genome had 204 SNP markers covering 1,830.03 cM with an average genetic distance of 8.97 cM between adjacent loci, while the Dt sub-genome possessed 300 SNP markers that span 2,585.97 cM with an average of 8.62 cM between successive loci. Overall, the average marker distance among the 26 chromosomes was 8.76 cM (Table 4). The longest and most dense chromosome found in this study was chromosome 18 (395.48 cM) with 79 markers, and the shortest and most sparse was chromosome 02 (85.03 cM) with 2 markers (Table 4 and Figure 1). In the genetic map of chromosome 2, there were only two markers mapped, and 19 markers showing segregated distorted (SD) were removed from the map. Thus, there is a huge gap in chromosome 2. Besides chromosome 2, other small gaps were recorded in this genetic map due to the removing of SD markers. Therefore, the marker interval in each chromosome was ranged from 7.9 to 85.0 cM (Table 4 and Figure 1).

**TABLE 4:** Details on the distribution of markers on different cotton chromosomes and their intervals, total span length of each chromosome and average marker distance obtained from the linkage analysis of the intra-specific cross genetic map developed in this study using the RILs derived from MCU5 and TCH1218.

Chromosome Name assigned in this study	Subgenome^$^	Total Polymorphic Markers	Mapped Markers	Span Length (cM)	Average Marker Distance (cM)	Maximum interval (cM)
Chromosome 1	A1	28	4	71.59	17.90	42.8
Chromosome 2	A2	23	2	85.03	42.52	85.0
Chromosome 3	A3	54	34	161.72	4.76	42.8
Chromosome 4	A4	30	5	94.84	18.97	33.7
Chromosome 5	A5	68	19	174.76	9.20	46.4
Chromosome 6	A6	43	10	160.68	16.07	44.3
Chromosome 7	A7	68	13	186.35	14.33	36.4
Chromosome 8	A8	85	36	256.09	7.11	59.9
Chromosome 9	A9	28	4	46.80	11.70	24.5
Chromosome 10	A10	81	19	136.73	7.20	55.3
Chromosome 11	A11	65	28	229.87	8.21	71.2
Chromosome 12	A12	85	20	205.25	10.26	54.2
Chromosome 13	A13	45	10	20.32	2.03	7.9
Chromosome 14	D02	136	51	99.52	1.95	28.3
Chromosome 15	D01	113	11	193.41	17.58	43.3
Chromosome 16	D07	38	3	81.77	27.26	81.7
Chromosome 17	D03	58	17	157.00	9.24	33.3
Chromosome 18	D13	193	79	395.48	5.01	41.2
Chromosome 19	D05	180	27	295.51	10.94	66.1
Chromosome 20	D10	120	34	356.24	10.48	50.9
Chromosome 21	D11	157	14	198.55	14.18	54.1
Chromosome 22	D04	56	9	155.84	17.32	54.1
Chromosome 23	D09	93	14	145.62	10.40	40.2
Chromosome 24	D08	109	15	144.09	9.61	54.9
Chromosome 25	D06	121	19	227.33	11.96	36.8
Chromosome 26	D12	61	7	135.61	19.37	52.6
	Whole Genome		504	4416.00	8.76	

*^$^Subgenome specific chromosome number assigned in physical map of the Gossypium hirsutum var TM1.*

**TABLE 5:** Collinearity analysis of the genetic map developed in the current study with a physical map of TM-1 Genome NAU-NBI Assembly v1.1 and Annotation v1.1 database.

Chr. No.	Start Marker	End Marker	Start Position	End Position	Genome spanned length	Reference Genome Length	Genome Coverage Ratio %
1	AX-182255420	AX-176831701	18867792	64144410	45276618	99884700	45.32888
2	AX-176833436	AX-182263372	654170	81801974	81147804	83447906	97.24367
3	AX-182255032	AX-176835952	100110564	739012	99371552	100263045	99.11085
4	AX-180658688	AX-176883453	61159919	54211865	6948054	62913772	11.04377
5	AX-182257133	AX-182247753	36181121	35105647	1075474	92047023	1.168396
6	AX-176831949	AX-176837003	97642105	2036386	95605719	103170444	92.66774
7	AX-176831470	AX-182253823	38496330	13923960	24572370	78251018	31.40198
8	AX-176883288	AX-176843209	1504641	69420054	67915413	103626341	65.53875
9	AX-184973861	AX-176883249	67775337	22284	67753053	74999931	90.33749
10	AX-176840552	AX-176831733	96620691	97693381	1072690	100866604	1.063474
11	AX-180647705	AX-176832026	4677099	84970420	80293321	93316192	86.04436
12	AX-180649246	AX-182253328	10771492	73316583	62545091	87484866	71.49247
13	AX-182260961	AX-176836152	5610554	63625753	58015199	79961121	72.55426
14	AX-182256274	AX-176834067	21699401	1054243	20645158	61456009	33.59339
15	AX-176832383	AX-182254104	53405972	5873728	47532244	67284553	70.64362
16	AX-184972992	AX-182258457	34647136	42362762	7715626	46690656	16.52499
17	AX-182254951	AX-182256079	46985857	2291470	44694387	51454130	86.86258
18	AX-176883283	AX-180651176	4528742	2326058	2202684	61933047	3.556557
19	AX-182261786	AX-182231153	62964863	11752151	51212712	64294643	79.65316
20	AX-182254614	AX-182257459	54179535	43190244	10989291	55312611	19.8676
21	AX-182245558	AX-176883438	41594632	64808056	23213424	65894135	35.22836
22	AX-182254106	AX-182254849	23362715	47166308	23803593	50995436	46.67789
23	AX-182247423	AX-182255641	61540989	25421890	36119099	63374666	56.99296
24	AX-176882838	AX-182258490	25421890	5369465	20052425	66087774	30.34211
25	AX-182256313	AX-182254915	300	47760462	47760162	59109837	80.79901
26	AX-182256906	AX-176883149	54800530	55191917	391387	60534298	0.646554
				**Total**	**1027924550**	**1934654758**	**53.1322**

**TABLE 6:** Identification of co-localized, stable, and major QTL for the investigated traits in this study.

Trait	Chromosome Number	QTL	Trial	Flanking markers/QTL Interval	QTL position	LR	Additive	Dominant	Main effect	PVE%	LOD
BN	5	*qBN-c05-1*	T3	AX-182253211-AX-182247753	158.11	12.3	−0.7608	0.6414	−0.84306	81.34	7.5
BW		*qBW-c05-2*	T4	AX-182253211-AX-182247753	158.11	12.3	−0.7608	0.6414	−0.84306	81.34	2.3
PH	9	*qPH-c09-1*	T1	AX-184973861-AX-176833420	20.01	16.25	−1.1294	−36.8499	32.62786	1.46	2.2
SYM		*qSYM_S-c9-1*	T6	AX-182229530-AX-176883249	31.41	21.32	4.609	−1.278	−0.27728	25.89	2.2
SYM		*qSYM-c09-1*	T4	AX-182229530-AX-176883249	33.41	36.25	−67.663	−3.5229	0.052065	47.56	5.3
PH	11	*qPH-c11-1*	T3	AX-176836296-AX-182226957	51.11	87.89	−47.3789	41.377	−0.87332	38.42	18.3
SYM		*qSYM-c11-1*	T4	AX-176836296-AX-182226957	58.11	36.52	21.2507	94.8709	4.464366	1.21	NA
SYM	15	*qSYM_S-c15-1*	T6	AX-176835896-AX-182239815	118.61	14.57	2.3089	−2.6836	−1.16229	8.99	2.4
SYM		*qSYM-c15-1*	T3	AX-182239815-AX-182262522	126.81	54.63	−20.5227	−71.0843	3.463691	23.35	9.6
SYM		*qSYM-c15-2*	T4	AX-182239815-AX-182262522	127.81	58.31	−28.2249	−91.8664	3.2548	22.00	9.1
SYM		*qSYM-c18-1*	T4	AX-182256182-AX-176832479	291.41	47.34	−28.6191	94.3655	−3.29729	87.39	8.6
SYM	18	*qSYM-c18-2*	T4	AX-182256182-AX-176832479	299.41	47.16	−68.1079	1.9272	−0.0283	87.84	8.6
SYM		*qSYM_S-c18-1*	T7	AX-182256182-AX-176832479	306.41	15.29	8.7063	3.5054	0.402628	6.11	3.6
SYM		*qSYM_S-c18-2*	T7	AX-176835242-AX-182255051	312.61	15.7	8.8199	3.4573	0.391989	6.00	3.6
SYM		*qSYM-c18-3*	T4	AX-176835242-AX-182255051	319.61	55.48	−68.3815	1.5155	−0.02216	87.35	8.6
SYM	21	*qSYM-c21-1*	T4	AX-182262518-AX-176832011	52.41	8.5	39.6384	0	0	8.47	1.6
SYM		*qSYM_S-c21-1*	T6	AX-182255439-AX-176832011	55.41	14.11	−6.8642	4.7052	−0.68547	67.95	3.6
SYM	22	*qSYM-c22-1*	T3	AX-182254626-AX-182258142	42.11	46.71	−51.8581	−1.0352	0.019962	80.33	NA
SYM		*qSYM-c22-4*	T3	AX-182254626-AX-182258142	44.11	57.51	−29.7937	−92.3846	3.10081	29.64	8.6
SYM		*qSYM-c22-2*	T3	AX-182254626-AX-182258142	47.11	54.42	−21.1143	−70.07	3.318604	25.73	NA
SYM		*qSYM_S-c22-1*	T6	AX-182254626-AX-182258142	54.11	11.4	5.8566	−1.1561	−0.1974	21.07	3.6
SYM		*qSYM-c22-3*	T3	AX-182258142-AX-182256858	62.11	49.24	−18.2436	−69.9692	3.835274	14.15	NA
SYM		*qSYM-c22-5*	T3	AX-182258142-AX-182256858	63.11	46.82	−25.2317	−90.2036	3.575011	7.07	8.6
SYM		*qSYM_S-c22-2*	T7	AX-182256858-AX-182264770	82.81	18.16	−8.2598	5.1913	−0.6285	88.02	3.6
BW	24	*qBW-c24-1*	T6	AX-182258056-AX-176831424	81.31	17.94	−0.2815	−1.0112	3.592185	7.14	NA
SYM		*qSYM_S-c24-1*	T6	AX-176834369-AX-182254666	109.71	27.77	10.583	1.7828	0.168459	26.26	3.5
PH	26	*qPH-c26-3*	T3	AX-182254129-AX-182226747	55.91	91.77	−47.1439	43.6916	−0.92677	0.02	18.5
BN		*qBN-c26-1*	T6	AX-182226747-AX-180652868	110.51	34.83	−2.5734	8.3187	−3.23257	83.62	7.9

*QTL nomenclature: qPH-c08-1 can be interpreted as: first letter “q” to represent QTL; second two letters to represent trait name (PH for plant height, SYM for number of sympodial, BN for number of bolls and BW for boll weight) under irrigated conditions (if there is _S suffix after the trait name), it means that the given trait was identified under water stress conditions; hyphen followed by “c” and a numeral denotes the chromosome number (chromosome 8 in the above case) and last numeral prefixed with a hyphen indicates the nth number of QTL identified for the given trait in the given chromosome. NA- not available.*

### High-Level Synteny, Collinearity, and Recombination Hotspot

**FIGURE 2:**
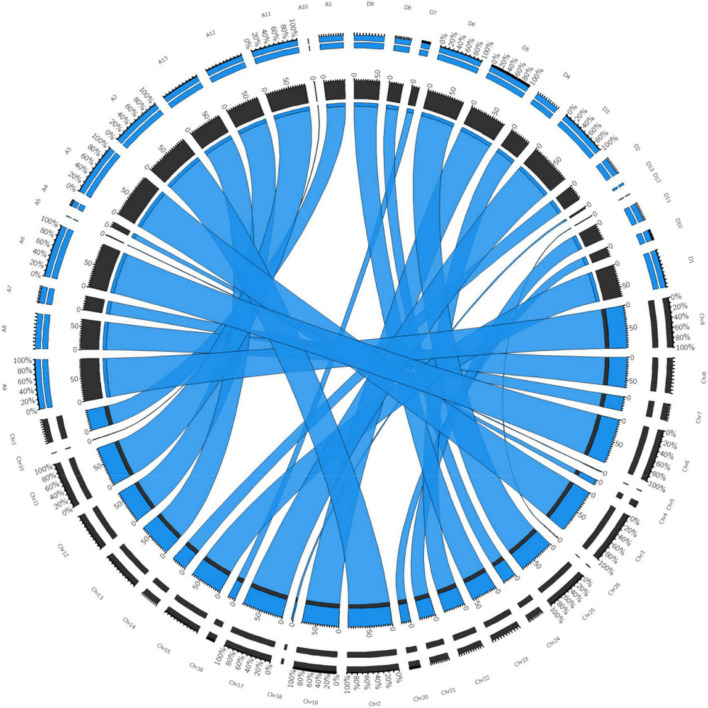
Collinearity of the TM-1 sequence based physical map (https://www.cottongen.org/analysis/251) corresponding to SNP-based genetic map constructed in this study. Chr1–Chr26 (represented in black color segment) are chromosome number of intraspecific map developed in this study. A01–A13 and D01–D13 (represented in blue color segment) are physical map of TM-1 taken from the CottonGen public database. Inner black (genetic map of present study) and blue segment (physical map of TM-1 from CottonGen) represents the total percentage of similarity, blue color ribbon represents co-linearity among homologous chromosomes, outer double segment represents the relative percentage contribution of chromosomes being compared.

The quality of the genetic linkage map was evaluated using parameters, such as the segregation distortion of mapped markers, gaps in the map, and collinearity between the linkage map constructed in this study and the reference physical map of TM-1^6^. All the 504 polymorphic SNPs found in this study were aligned to the *G. hirsutum* var. TM-1 reference genome using BWA in Galaxy and a high level of collinearity was shown by the SNP genetic map reported in this study with the physical map of the TM-1. It was revealed that there were no chromosomal rearrangement in the mapping population with context to the parents. The CIRCOS plot further confirmed the accuracy of the genetic maps (Figure 2). All the synteny blocks of each chromosome corresponded to the physical map and no single synteny block mismatched with the corresponding chromosome. Thus, it can be inferred that there were no chromosomal rearrangements. The sequence-based reference genome of 1,027.9 Mb corresponded to our SNP based genetic map of 4,416 cM and our genetic map represented 53.13% of the total length of the sequence-based physical map (Table 5). A good collinearity was revealed by all the linkage groups with the physical reference map (Figure 2). The coverage of individual chromosome in the constructed genetic linkage map of our study ranged from 0.64 to 99.11% of the physical reference map (Table 5). Chromosome 26 was least covered whereas, chromosome 3 was comprehensively covered. Though least covered chromosomes in the genetic map would provide little information during QTL analysis, it would be useful to have such preliminary data to proceed further.

### Quantitative Trait Loci for Productive Traits Under Irrigated and Water-Stress Conditions

As the variation found in the investigated traits was normally distributed (Table 2), it was predicted that multiple genes might control these traits. There was low to moderate heritability noticed for the investigated traits (Table 2). Thus, higher similarity between parents and offspring for the investigated trait was not expected making the QTL study more complex, and interesting for significant environmental influence. In such context, the identification of co-localized QTLs across the seasons and environments was the only means of solid proof for the presence of QTL linked to the investigated trait. A total of 53 QTLs for four investigated traits identified in this study, and among them, 21 and 32 were found to be At sub-genome- and Dt sub-genome-specific, respectively (Supplementary Table 1). The LOD values of all the identified QTLs ranged from 2.0 to 18.5. There were eight water stress-specific QTL further linked to different investigated traits. Though there are 8 QTL identified for PH (6 under irrigated conditions and two under water stress conditions), only two QTL had shown more than 10% phenotypic variation (Supplementary Table 1). Among them, *qPH-c11-1* explained 38.41% phenotypic variation (PVE) and was detected under irrigated conditions; whereas qPH_S-c16-2 was detected under rainfed conditions had 19.39% PVE. A maximum number of 36 QTL were detected for the number of sympodial branches (SYM), and among them, 25 QTL were detected under irrigated conditions, and 11 were detected under managed water stress/rainfed conditions. In these, QTL detected on chromosome 18 (*qSYM-c18-2*) had maximum phenotypic variation under irrigated conditions (87.83%) with QTL LOD of 8.6 and a QTL observed on chromosome 21 (*qSYM_S-c22-2*) had accounted for a maximum of phenotypic variation under rainfed conditions (88.01%) for SYM with QTL LOD value of 3.6. Seven QTL were also identified for boll number (BN) in which all the QTL were found under irrigated conditions. Among, *qBN-c26-1* exhibited 83.61% phenotypic variation. Only two QTL were found to be linked with boll weight (BW) under irrigated conditions, and no QTL was observed for this trait under rainfed conditions. The maximum PVE (81.34%) for this trait was reported for *qBW-c05-2*.

Twenty QTL had a positive additive effect for the investigated traits suggesting the independent effects of alleles on the trait (Supplementary Table 1). Several QTL were found to have a dominant positive effect (Supplementary Table 1). Among the 53 QTL, 25 QTL were derived from MCU5, which exhibited a positive additive effect, and 28 QTL were derived from TCH1218, which exhibited a negative additive effect. As expected, all the QTL detected under water stress conditions were contributed by the tolerant drought parent, TCH1218 (Supplementary Table 1) except *qPH_S-c16-1, qSYM_S-c18-1*, and *qSYM_S-c18-2*, which were contributed by drought susceptible parent, MCU5.

It was found that nine QTL that were not only co-localized for more than one investigated trait but also stable (identified at least in two seasons/locations) and major QTL with >10% PVE for at least one trait that colocalized in that region (Table 6). In addition, the maximum number of seven QTL was clustered on chromosome 22 and a minimum number of two QTL was found on chromosomes 5, 21, and 24 (Table 6 and Figure 1).

Further, joint multi-environmental QTL mapping effort to estimate the impact of QTL × Environment interaction resulted in the identification of 11 additive QTL (Table 7). Eight QTL associated with SYM were identified with LOD ranging from 3.02 to 5.70. The contribution rates of interaction among eight additive and the environment QTL ranged from 0.68 to 3.33%. Two QTL associated with PH were identified with LOD ranging from 3.13 to 3.53. The contribution rates of interaction among two additives and the environment QTLs ranged from 2.16 to 4.20%. A single QTL associated with BN was identified with LOD 4.36 and the contribution rates of interaction was 4.08% (Table 7). However, no QTL for BW was detected in this joint multi-environmental QTL analysis.

**TABLE 7:** Identification of Additive × Environment interaction effect QTL for the traits investigated in this study.

QTL	Chr	Position (cM)	Left Marker	Right Marker	LOD	LOD (A)	LOD (AbyE)	PVE	PVE (A)	PVE (AbyE)	Add	Aby E_01	Aby E_02	AbyE_03	AbyE_04
*qSYM_E-c11-1*	11	71	AX176836296	AX182226957	3.0249	0.0693	2.9556	0.193	0.0368	0.1562	−0.5344	1.2468	0.0854	−1.7606	0.4284
*qSYM_E-c11-2*	11	229	AX176882583	AX176832026	3.8848	0.5504	3.3345	0.6679	0.2792	0.3887	1.4646	−0.1463	−1.1551	2.8578	−1.5565
*qSYM_E-c15-1*	15	193	AX184973580	AX182254104	3.4658	1.037	2.4288	1.6958	0.5437	1.1521	2.0416	−1.3713	−1.6145	5.1253	−2.1395
*qSYM_E-c18-1*	18	15	AX182258223	AX182263818	3.9654	0.9728	2.9926	1.5777	0.5215	1.0561	1.9907	−0.9159	−1.7665	4.8443	−2.1619
*qSYM_E-c18-2*	18	53	AX182263818	AX176833896	3.0838	0.2411	2.8427	0.4394	0.1281	0.3113	−0.9875	0.1885	1.1967	−2.5703	1.1851
*qSYM_E-c24-1*	24	110	AX176834369	AX182254666	5.7394	5.0031	0.7363	10.1074	2.7141	7.3933	−4.5951	4.1812	4.3165	−13.1329	4.6352
*qSYM_E-c24-2*	24	144	AX180652387	AX182265405	3.0536	0.023	3.0306	0.1509	0.0124	0.1386	−0.3094	1.3595	−0.0955	−1.5376	0.2736
*qSYM_E-c26-1*	26	135	AX182226747	AX180652868	3.1082	2.4252	0.683	4.5312	1.2602	3.271	−3.1001	2.5187	2.9475	−8.6412	3.175
*qPH_E-c9-1*	9	41	AX182229530	AX176883249	3.1305	0.9694	2.1611	1.533	0.4049	1.128	−1.403	0.6183	−3.3961	−0.3659	3.1438
*qPH_E-c24-1*	24	144	AX180652387	AX182265405	4.5398	0.3374	4.2024	3.4359	0.192	3.2439	−0.9677	3.2493	−6.6381	0.4836	2.9052
*qBN_E-c19-1*	19	6	AX182255559	AX176830886	4.3675	0.2868	4.0807	1.8161	0.1695	1.6466	−0.1925	−0.8195	−0.004	−0.0513	0.8748

*QTL nomenclature is as that of Table 6 and the alphabet E denotes joint the multi-environmental QTL mapping. LOD: Logarithm of the Odds; A by E: Additive QTL × Environment; PVE: % of phenotypic variation explained.*

### Identification of Candidate Genes Within the Quantitative Trait Loci

Large array of candidate genes were identified within the QTL reported in this study (Supplementary Table 2), and they were compared and annotated with Xu et al. (2020), where meta-QTL analysis along with transcriptomic approach utilized for the identification of candidate genes related to fiber quality in upland cotton. Though there were genes specific to abiotic stress responses and productive traits, large numbers of genes identified in this study warrant the use of additional markers to fine map these QTL and identify precise genes involved for the target traits. For example, for the PH QTL under irrigated conditions, *qPH-c11-1*, there were 32 genes, whereas under water stress conditions, the QTL identified for PH, *qPH_S-c16-2*, possessed 94 candidate genes (Supplementary Table 2). Similarly, the QTL identified for a number of sympodial branches underwater-stressed environment, *qSYM_S-c24-1* harbored 169 genes and QTL for BN under irrigated conditions, *qBN-c26-1* contained 733 genes, and the BW QTL under irrigated conditions, *qBW-c05-2* had 2,074 genes.

The GO analysis used all the identified candidate genes to identify potential biological functions and grouped under three main GO categories: biological process, molecular function, and cellular component (Supplementary Table 3; Sheet “GO”). Within the biological process category, there were 12 sub-categories, such as abiotic stress-specific, such as response to freezing, and response to biotic stimulus sub-category possessed the maximum number of 15 candidate genes. ADP binding with 21 genes was the main sub-category in the molecular function category with 14 principal sub-categories. Finally, in the cellular component category, there was one sub-category (vacuolar membrane) with 11 genes (Supplementary Table 3; Sheet “GO”). On the other hand, KEGG analysis resulted in only one category, namely, plant-pathogen interaction with four candidate genes (Supplementary Table 3; Sheet “KEGG”).

## Discussion

Some cotton lines have developed unique tactics to successfully handle the challenges of shifting and unexpected conditions, particularly under water stress. However, due to the intricacy of these characteristics and a lack of knowledge of the genetic processes behind these traits, introducing such drought tolerance qualities into elite cotton cultivars through conventional breeding approaches has been sluggish. We used an intraspecific linkage map generated using SNP markers to identify QTL connected to the productive attributes under water stress in this work. RILs developed from a cross between MCU5 (a well-known commercial cultivar in South India) and a good combiner, TCH1218, were tested in the field under irrigated, managed water stress, and rainfed conditions to identify QTL conferring productive traits. The RILs used in this study are useful and valuable asset for the QTL mapping of productive traits under water-limited environments. The individuals of RILs exhibited almost all possible kinds of variations for the productive traits under water stress (Table 2).

It was noticed that water stress invariably reduced the productive trait expression in all the trials and the impact of drought included the wilting and drooping of leaves and reduced boll set and ultimately worsened the yield. Similar kinds of the impact of water stress in cotton have already been reported (Saranga et al., 2004; Zheng et al., 2016). The broad-sense heritability for the investigated traits was low to moderate (Table 2), and it indicated that the drought tolerance in cotton is greatly influenced by multiple genes and strongly affected by the environmental conditions. The impact of environmental conditions on the productive traits has already been reported in cotton (Jia et al., 2014). Though those low to moderate heritability may not accurately predict QTL, it may specify the fact that the investigated planting materials experienced relatively uniform treatments in all the trials. Furthermore, individual RILs in this work demonstrated a wide variety of reactions to the features under consideration (Table 2), and it was discovered that such responses are reasonably constant between trials. This study, in addition to having the advantage of screening the RILs under real field stress conditions with appropriate replications, also provides an example of how to carry out the experiment with less expenditure and more realistically when compared with artificial screening for drought tolerance in cotton.

This would be more useful in this genomics era, as genotyping costs are increasingly reduced, but phenotyping costs are increased tremendously when advanced and complicated infrastructure is developed for drought resistance screening. Thus, this study has shown that the replicated field screening using permanent mapping populations, such as RILs, under natural water stress conditions in multiple target environments provide valuable information for accurate QTL mapping in crop plants, such as cotton (Boopathi, 2020). Further, the positive correlation reported in this study among the examined traits specified that productive traits can be improved by selecting the agronomical traits, such as appropriate PH and the number of sympodial branches.

### Single Nucleotide Polymorphisms for Linkage Map Development

The lack of large numbers and the segregation distortion of polymorphic markers are the main leading cause for the availability of a limited number of high-density linkage maps in cotton (Zhang T. et al., 2015). As a result, cotton genetic maps with SSR or AFLP markers often shows large gaps, poor marker density, and low marker coverage, whereas SNPs have shown their utility in constructing a high-dense genetic map as they are distributed throughout the genome (Cai et al., 2017; Liu et al., 2018). Among various strategies used in SNP genotyping (such as, genotyping by sequencing and whole-genome resequencing), SNP arrays have been shown to be simple and useful in the development of dense genetic maps in cotton (Li et al., 2016; Tan et al., 2018; Kumar et al., 2019). For example, in the present study 2,457 polymorphic markers between the parents of an intraspecific cross of upland cotton were identified using Cotton SNP50K array.

Progenies resulting from various cotton cross combinations frequently have unequal allelic distribution (Shao et al., 2014). Because of marker and population types, preferred fertilization, gametic combinations, genetic drift, and environmental variables, segregation distortion can vary across and within species (Shen et al., 2007). Despite the fact that a considerable number of polymorphic markers were found in this study, a large proportion of them (1,953, or 79.48% of total polymorphic loci identified between the parents) were removed due to segregation distortion. As a result, the number of loci in this study that can be adequately mapped has been drastically decreased. Cotton has also shown a low degree of mapping effectiveness for discovered polymorphic markers (Lacape et al., 2010; Yu et al., 2013). On the other hand, the constructed genetic map in present study did not have any segregation distortion region.

Thus, after a stringent screening of missing data, segregation distortion, and similar loci, a total of 504 markers were mapped only on 26 chromosomes. As shown in Table 4, the highest number of markers (300 SNPs) was distributed on Dt sub-genome when compared with At sub-genome (204 SNPs). It was inferred that the genetic map constructed in this effort had highest collinearity with the physical map (all the 504 polymorphic SNPs were aligned perfectly; Figure 2) and had covered 53.13% of the physical map (TM-1 Genome NAU-NBI Assembly v1.1 and Annotation v1.1 database; Table 5), which highlighted that there was no chromosomal rearrangement in the intraspecific genetic map constructed in this study.

### Quantitative Trait Loci for Productive Traits Under Irrigated and Water Stress Conditions

Several small effect QTL are involved in confirming the drought tolerance in cotton, and each loci represents hundreds of genes, which are the genetic basis for expressing an extensive array of water stress responses in the form of morpho-physiological traits (Abdelraheem et al., 2021). As only a few studies have focused on the QTL mapping of drought tolerance in cotton (reviewed in Mahmood et al., 2020), the molecular breeding of cotton for the genetic improvement of water stress tolerance is considered as a challenging task. Further, such QTL have large intervals (Mahmood et al., 2020; Abdelraheem et al., 2021).

This study found large intervals in the identified QTL, mainly attributed to the poor polymorphism rate detected between the two parents used in this study. Further, due to the segregation distortion, the number of mappable markers was reduced drastically from 2,457 to 504, which resulted in due to the segregation distortion. This drastic reduction of mappable markers resulted in gaps in the genetic map constructed in this study, reporting in *G. hirsutum* (Wang et al., 2006; Wu et al., 2009; Sun et al., 2012) and even in interspecific crosses of *Gossypium* spp. (He et al., 2007; Lacape et al., 2010).

Though there are different strategies (such as, genotyping by sequencing) to increase the efficiency of genetic mapping and QTL analysis through fine mapping, it would be desirable to employ high-density SNP chips as it enables speedy and automated detection of a high rate of polymorphisms across cotton accessions within a short span of time. Therefore, the development of new SNP chips by using an additional set of SNPs, detected on the water-stress responsive candidate genes identified in this study, would further enhance the efficiency of cotton molecular breeding for abiotic stress resistance.

Compared with the published reports on QTL linked to the drought tolerance and fiber yield and quality traits (Mahmood et al., 2020), a relatively low number of QTL were detected in this study. It indicated that the traits examined in this study were under strong genotype by environment (GxE) interaction and low heritability. Despite this, nine common QTL were detected across the seasons and locations, which pointed out that the collected phenotypic data are reliable even though there were GxE interactions. Further, a significant correlation among those traits and a substantial amount of heritability and additive effect estimates for most of the QTL identified in this study showed that the selection of investigated traits for drought tolerance improvement in cotton under filed water stress conditions would be more efficient. Similar evidence for selecting productive traits under abiotic stress environments in cotton has already been reported (Abdelraheem et al., 2018, 2021).

Surprisingly, both parents contributed to the additive effects of water stress tolerance alleles (Table 6). This demonstrated that even the inferior parent (MCU5) might contribute to the establishment of drought tolerance, a feature that has previously been demonstrated (Paterson et al., 2003; Ulloa et al., 2005; Shen et al., 2007). It is imperative to identify the consensus and hot spot QTL across the testing environments (as shown below), which will facilitate the introgression of causal genomic regions that impart at least a minimum increase in the productivity under water stress. Though this study had identified several QTL (Supplementary Table 1), only those QTL that have shown their potential in molecular breeding have been selected (Table 6) for further analysis. This study had identified a total of 11 QTL in joint multi-environmental analysis. Compared with individual analysis (Supplementary Table 1), the joint multi-environmental analysis (Table 7) identified a smaller number of QTL due to both QTL additive and QTL-environment interaction effects. However, the former analysis estimates the only additive effect of QTL but not the environmental influence. Thus, this effort has helped to get additional information on the influence of the environment on the expression of QTL under different water-limited environments.

### Hotspot Quantitative Trait Loci That Colocalized for Multiple Traits and Candidate Genes

Further, this study attempted to identify hotspot QTL (which is defined here as a cotton genomic region), where QTL were discovered for more than one trait that was investigated across the seasons and locations expressing with more than 10% phenotypic variations (in this study) and genetic backgrounds (elsewhere) were colocalized in the same genomic segment. Nine hotspot QTL were identified in this study that were located on chromosomes 5, 7, 9, 11, 15, 18, 21, 22, 24, and 26 (Table 6). Among them, a region on chromosome 22 flanked by AX-182254626–AX-182264770 with a span length of 89.4 cM, is co-localized for a maximum number of major and stable QTL (7) linked to the number of sympodial branches both under irrigated and water stress conditions (Figure 1). This region was specifically detected for fruit branch node number by Hulse-Kemp et al. (2015). Another notable region was on chromosome 18 that span a length of 61.4 cM flanked by the SNPs, AX-182256182 and AX-182255051. This region was associated with five QTL linked to the number of sympodial branches both under irrigated and water-stressed conditions in this study. Similarly, a QTL cluster was found on chromosome 5 for BW and BN in this study and similar kind of correlation of this chromosomal region with several productive traits has been reported earlier (Abdelraheem et al., 2018).

Except for chromosomes 5 and 26, almost all of the hotspots discovered in this study were connected to the number of sympodial branches. Cotton bolls can form at the nodes of sympodia that develop from the monopodia, as well as at the main or secondary vegetative axis sites. Orderly development of bolls on sympodia has long been favored (McClelland, 1916), and plant design with 0% monopodial growth would be more ideal, favoring efficient mechanical harvesting and improving ultimate yield. Since only major, stable, and consistent QTL were reported in this study, it is likely that all of the QTL listed in Table 6 could be potential assets for consensus mapping across genetic backgrounds for their validation and identification of most useful QTL hotspots, map-based cloning, and MAS for productive traits in upland cotton with high predictability. Among the three PH QTL chosen in this investigation (Table 6), a QTL on chromosome 11, qPH-c11-1, has demonstrated the most phenotypic variance (38.41%), and this area has been proven to host PH QTL under irrigated (Jia et al., 2016) and salt stress environments (Abdelraheem et al., 2021). Optimum plant height in cotton is the most desirable trait in the terms of mechanical harvesting, besides its direct relation with biomass that influences the final yield (Shang et al., 2015).

It would be an elaborated list to identify candidate genes for the QTL reported in this study, as it has been estimated that ∼4,500 cM of *G. hirsutum* genome consisted of 70,478 predicted protein-coding genes (Zhang T. et al., 2015). Similarly, the large array of candidate genes was found in this study (Supplementary Table 2). Despite this extensive list, identifying candidate genes in the target hotspot QTL can open up new avenues in understanding the molecular basis of drought tolerance in cotton.

For example, *qSYM-c22-1*, a QTL on chromosome 22 flanked by AX-182254626 and AX-182258142, was found to be co-localized for the number of sympodial branches both under irrigated and water-stressed conditions, and it harbors 1,046 genes (Supplementary Table 2). Among them, the notables are GDSL esterases/lipases, involved in the regulation of plant development by the synthesis of secondary metabolites in response to the biotic and abiotic stresses (Chepyshko et al., 2012), zinc finger protein CONSTANS, regulating flowering under normal and stress conditions (Putterill et al., 1995), abscisic acid receptor PYL8, expressed in response to dark-induced leaf senescence (Lee et al., 2015), choline mono oxygenase, conferring abiotic stress tolerance by synthesizing glycine betaine (Bao et al., 2011), potassium transporter 2, copper transport protein ATX1, and peroxidase 52, contributing significantly for abiotic stress resistance (Zhang et al., 2021) and several network of reactive oxygen species (ROS) genes, those have played critical role in both abiotic stress response and fiber development (Xu et al., 2019). Similar kinds of candidate genes were identified for another hotspot QTL (Supplementary Table 2). Thus, this study provides a promising lead to precise the QTL mapping consisting of functional SNPs derived from those candidate genes identified in the target QTL by developing a new SNP chip.

## Conclusion

Identifying major and stable QTL for productive characteristics in water-stressed conditions is a precondition for developing an effective cotton molecular breeding program. This work found such QTL using intraspecific RILs, which allowed them to be examined in multiple seasons and circumstances. Collecting replicated phenotypic data for successful QTL mapping requires intraspecific RILs, which allowed them to be evaluated in different seasons and situations. Though this study contributed preliminary information on candidate genes that unravel the molecular mechanism underlying cotton productivity under water stress, fine-mapping those QTL using additional SNPs derived from those candidate genes would be required to validate and employ them in MAS for the genetic improvement of cotton with improved productivity under water-stressed environments.

## Data Availability Statement

The datasets presented in this study can be found in online repositories. The names of the repository/repositories and accession number(s) can be found in the article/Supplementary Material.

## Author Contributions

NB and SJ designed the experiments, prepared the documents for funding, and drafted the full manuscript. NB developed the RIL reported in this study. GT and SJ performed all the analyses of genetic and QTL mapping. NB, KN, PS, VS, NP, and SR evaluated mapping populations at different locations and years and collected phenotypic data on fiber traits. GT and BJ drafted the results sections and edited the manuscript. All authors contributed to the article and approved the submitted version.

## Conflict of Interest

The authors declare that the research was conducted in the absence of any commercial or financial relationships that could be construed as a potential conflict of interest.

## Publisher’s Note

All claims expressed in this article are solely those of the authors and do not necessarily represent those of their affiliated organizations, or those of the publisher, the editors and the reviewers. Any product that may be evaluated in this article, or claim that may be made by its manufacturer, is not guaranteed or endorsed by the publisher.

## Footnotes

1
https://www.worldatlas.com/articles/top-cotton-producing-countries-in-the-world.html

2
https://cotcorp.org.in/national_cotton.aspx

3
https://www.abs.gov.au/media-centre/media-releases/australias-cotton-production-37-year-low

4
https://www.calculatorsoup.com/calculators/statistics/descriptivestatistics.php

5
https://www.minitab.com/en-us/support/downloads/

6
https://www.cottongen.org/analysis/251
